# Evidence-based practice implementation: The impact of public versus private sector organization type on organizational support, provider attitudes, and adoption of evidence-based practice

**DOI:** 10.1186/1748-5908-4-83

**Published:** 2009-12-31

**Authors:** Gregory A Aarons, David H Sommerfeld, Christine M Walrath-Greene

**Affiliations:** 1 Department of Psychiatry, University of California, 9500 Gilman Drive #8012, San Diego, CA 92093-0812, USA; 2 ICF Macro 116 John Street, Suite 800, New York, NY 10038, USA

## Abstract

**Background:**

The goal of this study is to extend research on evidence-based practice (EBP) implementation by examining the impact of organizational type (public versus private) and organizational support for EBP on provider attitudes toward EBP and EBP use. Both organization theory and theory of innovation uptake and individual adoption of EBP guide the approach and analyses in this study. We anticipated that private sector organizations would provide greater levels of organizational support for EBPs leading to more positive provider attitudes towards EBPs and EBP use. We also expected attitudes toward EBPs to mediate the association of organizational support and EBP use.

**Methods:**

Participants were mental health service providers from 17 communities in 16 states in the United States (n = 170). Path analyses were conducted to compare three theoretical models of the impact of organization type on organizational support for EBP and of organizational support on provider attitudes toward EBP and EBP use.

**Results:**

Consistent with our predictions, private agencies provided greater support for EBP implementation, and staff working for private agencies reported more positive attitudes toward adopting EBPs. Organizational support for EBP partially mediated the association of organization type on provider attitudes toward EBP. Organizational support was significantly positively associated with attitudes toward EBP and EBP use in practice.

**Conclusion:**

This study offers further support for the importance of organizational context as an influence on organizational support for EBP and provider attitudes toward adopting EBP. The study demonstrates the role organizational support in provider use of EBP in practice. This study also suggests that organizational support for innovation is a malleable factor in supporting use of EBP. Greater attention should be paid to organizational influences that can facilitate the dissemination and implementation of EBPs in community settings.

## Background

Policy and practice directives emphasize improving service quality and effectiveness in mental health services through the development, dissemination, and implementation of evidence-based practices (EBPs) [1,2]. However, the recent proliferation of promising and empirically tested interventions and protocols has not been matched by widespread and effective implementation of such practices in community settings. Concern about this 'knowledge-practice' gap has focused attention on identifying and testing mechanisms that facilitate or inhibit EBP dissemination and implementation [3-5]. Theory and research indicate that the adoption and use of EBPs is influenced by both organizational context and individual adopter characteristics [4,6]. In keeping with the notion of multiple determinism, the present study focuses on relationships between characteristics of organizational context and provider characteristics. Specifically, we examined the relationships among organization type (public versus private for profit), organizational support for EBPs, clinician attitudes towards adopting EBP, and EBP use.

Our examination of these relationships is informed by empirical research on institutional theory [7] and the theory of planned behavior [8] to provide a general guiding framework. The institutional approach posits that organizations, and the individuals within them, are shaped by social norms and expectations, and that organizational structures and behaviors typically conform to their social, legal, and technical environments [9]. An implication of this perspective is that organizations of different types, defined here as either public (*e.g.*, government) or private (*e.g.*, for-profit or nonprofit) agencies and their staff, are likely to exhibit systematic structural and behavioral differences relevant to adoption and implementation of EBPs. Building on our prior empirical research [10], this study examines the relationship between organizational type and organizational support. We also examine the association of both organizational type and organization support for EBP with clinician attitudes toward EBPs. We raise the issue and test whether organization support for EBP is associated with higher levels of EBP use in practice and more positive attitudes toward adopting EBP.

Additionally, the theory of planned behavior contributes to our expectation that attitudes toward adopting EBP will be related to EBP use in practice. In the theory of planned behavior, [8] an individual's attitudes regarding a specific behavior represents an important component in determining whether a specific behavior will be enacted. Following theory that attitudes precede behavior, our study examines whether attitudes towards EBP are associated with EBP use.

This study contributes to the implementation science literature because minimal research has explicitly examined the relationship between organizational type and the adoption and implementation of EBP despite the fact that many health and human service industries are comprised of a mixture of government, for-profit, and nonprofit entities [11,12]. Also, this study examines the relationship organizational support for EBP, attitudes towards EBP, and the use of EBPs in practice. Identifying such relationships would provide evidence that efforts to improve organizational support can impact both attitudes and use, representing important mechanisms for increasing the adoption of EBPs. In the following sections, we describe the theoretical underpinnings of the model to be tested in the current study.

### Provider attitudes toward EBP

There is increasing evidence that the values and beliefs of the individual adopter play an important role in the degree to which innovations are initiated and incorporated into common practice [4,6,13]. Such attention to innovation, adopter values, and beliefs highlights the importance of studying mental health service provider attitudes regarding the implementation of evidence-based service innovations. Service providers operate at the critical interface between health and mental health service delivery organizations, effective treatments, and the specific needs of individuals and families receiving services. The measurement of provider attitudes toward adopting EBPs has been facilitated through the development of the EBP attitude scale (EBPAS) [10]. Research utilizing the EBPAS has documented that both organizational factors and service provider characteristics are associated with attitudes toward adopting EBPs. As noted above, organizational factors are important in innovation implementation [6,14,15] and emerging research has demonstrated significant relationships between provider attitudes toward EBP and organizational characteristics. For example, organizational attributes such as less bureaucratic organizational structure, the presence of formalized practice policies, positive organizational culture and climate, and greater transformational leadership styles are associated with more favorable service provider attitudes toward adopting EBPs [10,16,17]. Individual service provider characteristics such as higher educational attainment and being earlier in one's career are also associated with attitudes toward EBP [10]. However, much remains unknown about the how organizational characteristics impact provider attitudes. Organization type is one factor that might influence clinician attitudes.

### Organization type and attitudes toward EBP

Institutional theory highlights the need for organizations to act, or at least give the appearance of acting, in a manner consistent with social norms and expectations in order to maintain their legitimacy and associated resource flows [7]. One manner through which this process occurs is the development of organizational structures that reflect the demands of the broader social, technological, and legal environment [9]. An imperative for public sector organizations is a heightened demand for public accountability, fairness, and uniformity [18]. This suggests that despite pressures to minimize variations between organizations operating within the same industry [19], public and private organizations will likely differ with public agencies relying more on mechanisms that signify fairness and accountability, such as the formalization of rules and development of bureaucratic structures [18]. The empirical literature supports the expectation that public sector organizations demonstrate greater bureaucracy and more formalization of rules, regulations, and hierarchical authority structures than private sector agencies [20].

The higher levels of bureaucracy found within public sector organizations has implications for innovation as greater perceptions of 'red tape' have been associated with more risk averse managers [21], and reduced risk tolerance has been linked with diminished interest in innovation and change [22]. These mechanisms help to explain why managers in government agencies have been found to be less entrepreneurial than their private sector counterparts [23]. Such sector differences likely contribute to the private sectors' early and more widespread adoption of EBPs within substance abuse treatment organizations [24,25]. Prior research has also found more favorable attitudes among mental health providers in non-governmental agencies relative to those working in governmental agencies within one large metropolitan county [10]. The current study attempts to test such a relationship using a geographically diverse organizational sample and also examining the extent to which organizational support for innovation plays a role in this process.

### Organization type and organizational support for innovation

Public and private sector agencies are likely to differ in the degree to which organizational support for EBP is present. Public sector agencies may be less likely to engage in innovation and change and, even if they endorse change, may try to implement change by 'command and control' rather than engaging in more facilitative and supportive change strategies [23]. Thus, regardless of the specific mechanism, it is likely that public sector agencies would provide fewer organizational supports for EBPs. If a systematic relationship between organizational type and organizational support of EBPs is identified, then organizational support may operate as a mediating influence on the relationship between organizational type and provider attitudes towards EBPs, or as a mediator between organization type and EBP use.

### Organizational support for innovation and attitudes toward EBP

The theory of perceived organizational support posits that employees' perceptions of an organization's commitment to staff will influence their work-related attitudes and actions [26]. Three forms of organizational support (*i.e.*, fairness, supervisor support, and organizational rewards and job conditions) have been associated with measures of perceived organizational support. Perceived organizational support has been subsequently related to work outcomes, including higher job satisfaction, improved performance, and, most pertinent to the present study, greater job involvement [27]. This theory and its broad empirical support highlight the capacity for organizations to directly influence employee work attitudes and behaviors through providing (or withholding) forms of organizational support.

### Organizational support for innovation and EBP use

In the context of implementation, research has identified the availability of organizational supports for innovation to be important for successful and effective implementation of innovation [3,5,28,29] and as an important component of a facilitative implementation climate [29]. In addition, the impact of organizational resource availability for EBP implementation and the extent to which support is voiced and offered may also provide a signal to employees about the overall endorsement or orientation of the organization towards EBP. If organizations provide a wide range of supports for EBP, then employees may perceive that EBPs are viewed as a desirable and even preferred approach to service provision and support may directly lead to behavior change. In contrast, if organizational supports are limited and not palpable throughout the organization, employees may be less likely to adopt an innovation such as an EBP. An empirically informed multi-level model of innovation adoption suggests that organizational facilitators of innovation, such as providing training and other forms of support, contribute to behavior change such as adopting an EBP.

### Organizational support, attitudes toward EBP, and EBP use

Previous research has shown that higher levels of management and organizational support for implementation are associated with implementation effectiveness [29]. However, the impact of organizational support on behavior may be mediated by employee attitudes. The theory of planned behavior has received substantial empirical support and overlaps with theoretical frameworks outlining the components needed for successful adoption and implementation of innovative behaviors within organizations by identifying attitudes as an influence in the adoption of and adherence to behavioral change [3,6]. While studies have linked organizational and individual provider characteristics to provider attitudes toward EBP, no studies have examined the link between organizational support for innovation, attitudes towards EBP, and subsequent use of EBPs. However, theory suggests that organizational support should impact attitudes, and that both organizational support for adopting EBP, and attitudes toward EBP may be associated with EBP use.

### The present study

The present study advances our understanding of how organization type impacts organizational support for EBP and provider attitudes toward adopting EBP. It also advances our understanding of the impact of organizational support for EBP on provider attitudes toward EBP and on EBP use in practice. First, compared to our earlier work [10,16,17] the present study uses a more geographically diverse national sample (16 states) of organizations to examine the association between organization type and provider attitudes toward EBP [10]. Second, this study extends previous research by examining the association of organization type with level of organizational support for EBP. Third, the present study explores whether the level of tangible organizational support for EBPs influences mental health provider attitudes toward adopting EBPs. Fourth, the study examines the direct effect of organizational support for EBP on use of EBP in practice. This focus helps illuminate a potential area for organizational interventions to improve EBP implementation. Finally, the present study examines links between organizational support for EBP, provider attitudes toward EBP, and EBP use. Examination of these issues has the potential to increase our understanding of EBP implementation and help inform implementation strategies within both governmental and private sector agencies. Based on prior theory and research we proposed the following hypotheses:

1. More positive provider attitudes toward EBP will be found in private versus governmental organizations,

2. Private sector organization type will be associated with higher levels of organizational support for EBP.

3. Higher levels of organizational support for EBP will be associated with more positive provider attitudes toward adopting EBPs.

4. More positive attitudes toward adopting EBPs will be positively related to EBP use.

5. The effect of organization type on provider attitudes toward EBP will be partially mediated by level of organizational support for EBP.

6. Organization support for EBP will be associated with EBP use.

7. There will be direct and indirect effects of organizational support for EBP on EBP use.

## Methods

### Sample identification and recruitment

The context for this study was in communities funded under the United States Federal Comprehensive Community Mental Health Services for Children and Their Families (CCMHS) Program [30]. Data collection methods were developed in conjunction with the CCMHS national evaluation, and data collection was conducted by a Macro International, Inc. evaluation team. A list of potential respondents was generated using snowball sampling [31] that involved structured community-contact telephone calls to 22 currently funded CCMHS communities to identify all of the local mental health agencies serving children with severe emotional disturbance. Identified agencies were asked to provide a list of their mental health service providers resulting in the identification of 703 potential respondents.

Next, a multi-stage emailing process [32] was utilized including: a pre-survey email; survey invitation email with web link, username, and password; reminder email to the full sample; reminder follow-up email; and targeted follow-up phone calls to non-responders. Data collection was conducted August through November 2005. The study was approved by institutional review boards at the organizations conducting the study, and respondents were informed that completion of the survey indicated their consent. Survey responses were received from 288 mental health providers representing a response rate of 41%, which is consistent with other published response rates for surveys of this type [33]. Data from respondents who did not complete all primary sections of the survey were excluded resulting in a study sample of 174 respondents from 17 different communities representing 16 states spanning the United States of America.

### Participants

All participants were direct providers of mental health services to children and families. Of these respondents, 106 (60.9%) worked for private-not-for-profit agencies, 42 (24.1%) worked in public mental health agencies, 24 (13.8%) worked for private-for-profit agencies, and the remaining 2 (1.1%) for 'other' types of agencies. Respondents had worked as child/family mental health providers for a mean of 9.65 years (SD = 7.89). Their mean age was 40.75 years (SD = 11.20; Range = 23-72), and 126 (72.4%) were female (data missing for one respondent). Twenty-three respondents (13.3%) had a doctoral degree, 120 (69.4%) a masters degree, 28 (16.1%) a bachelors degree, and 2 (1.1%) had attended some college but had no degree (data missing for one respondent). The respondents represented a range of academic disciplines including social work (n = 57, 32.8%), psychology (n = 44, 25.3%), counseling (n = 28; 16.1), marriage and family therapy (n = 11, 6.3%), and 'other' disciplines (n = 31, 17.8%; *e.g.*, education, nursing, *et al.*; data missing for 3 (1.7%) providers). Respondents primarily self-identified as Caucasian (n = 153; 87.9%; missing data for one respondent).

## Measures

### Provider demographics

Demographic variables included gender (male = 1), race (white = 1), age, agency tenure (years at agency), years in child mental health, and education level. Education was measured using a six-point ordinal scale ranging from high school diploma/GED to doctoral degree (Ph.D., M.D. or equivalent).

### Organization type

Organization type was identified based on survey responses. Participants were identified as working for either a private sector or a public sector (*i.e.*, government-operated) agency. Private sector agencies included those operating as either for-profit or nonprofit organizations.

### Organizational support for EBP

Organizational support was the sum of nine items addressing specific processes/structures supporting the use of EBP in the organization. Items were developed as part of a 2003 United States national evaluation of the implementation of mental health systems of care. The primary domains included in the original survey (*i.e.*, provider knowledge, perception and use of evidence-based treatments and practices, as well as provider training opportunities and organizational supports for the use of evidence-based treatments and practices) were identified through a review of the extant literature and by experts in the field of best practice treatment and its implementation. Data regarding organizational support for EBP were collected via an open-ended question: 'What specifically does your agency/organization do to support you in your efforts to provide evidence-based treatment when appropriate?' asked of over 450 direct mental health service providers. This data was thematically analyzed and categorized. Data reduction resulted in the nine discrete close-ended items included in the 2005 version of the survey upon which the current study is based [34]. In the present study, each item used a dichotomous (no = 0/yes = 1) response regarding supports provided by the respondent's agency within the past year to assist efforts to implement EBP. The nine items included: 1) agency sponsored EBP trainings or in-services; 2) conferences, workshops, or seminars focusing on EBP; 3) guest speakers presenting about EBP; 4) EBP specific supervision and/or general guidance from administrators; 5) continuing education and/or grand rounds focused on EBP; 6) agency conducts internal research and/or evaluation, provides data regarding EBP; 7) agency provides EBP training materials or journals; 8) provides time off or funding for individual training/education in EBP; and 9) agency provides financial incentives to use EBP.

In order to examine scale dimensionality, we conducted three factor analyses; one for the whole sample (n = 170, four cases had missing data, see Analyses section below), and one each for public (n = 41) and private (n = 129) agency clinicians separately. In order to determine the appropriate number of factors, we examined the scree plot, factor loadings, and interpretability for each solution. We found a clear unidimensional solution for the whole sample and for the private agency respondents. The sample size for public agency respondents was small, thus results may have been less stable for this group. However, the scree plot for the public agency solution also suggested a one-factor solution. While two items had loadings on a second factor, this factor was not readily interpretable in that the items each represented a different aspect of support for EBP (*i.e.*, financial incentives, internal research/evaluation). Thus, in consideration of the equivocal quantitative results and small sample size for public agency respondents, we accepted a unidimensional model for the present study. We also computed the Kuder-Richardson 20 internal consistency reliability for the scale and found a value of 0.81, indicating strong internal consistency for the measure of EBP support in this sample.

### EBP attitude scale (EBPAS) [10]

The EBPAS is a very brief 15-item measure that assesses mental health and social service provider attitudes toward adopting EBPs. The EBPAS has also been adapted for use in medical, social service, and school settings, and has be translated into Spanish, Japanese, Korean, Romanian, Swedish, and Norwegian. EBPAS items are rated on a five-point Likert scale ranging from 0 (Not at all) to 4 (To a very great extent). The EBPAS total scale score (used in the present study) represents respondents' global attitude toward adoption of EBPs. Cronbach's alpha reliability for the overall EBPAS is good (α = 0.79), with subscale alphas ranging from 0.93 to 0.66 [35]. The measure's validity is supported by associations with mental health clinic structure and policies [10], culture and climate [17], and leadership [16]. The EBPAS is available from the first author.

### Use of EBP

Providers were asked to identify which EBPs (from a list of 31 child and/or family focused interventions) they utilized during the past year with children and families participating in the systems of care program. Items for EBP use were developed in conjunction with the measure of organizational support for EBP described above [34]. Similar to prior research on EBP use [36,37]., the EBP use measure was constructed by summing the number of individual EBPs used by each provider. In order to provide a more conservative estimate of EBP use, the total count of EBP practices used only includes EBPs for which the provider reported receiving specific training in either graduate school, a conference/workshop, an agency in-service, or a continuing education program. The 31 EBPs are presented in Appendix 1.

### Analyses

We used path analysis because it allows testing of an *a priori *complex model while controlling for covariance of all study variables. In contrast to hierarchical regression models, path analysis allows more flexibility in specifying the relationships of variables in the model. Although some preconditions for assessing effects of mediation of organizational support on EBP by provider attitudes were not met based on the relationships presented in the correlation matrix [38], hypotheses were directly explored in the path models below. Additional rationale for conducting path analysis in the absence of traditional criteria includes low power and Type I error rates that are too conservative relative to other approaches to testing mediation, such as the distribution of the product method [39].

We used maximum likelihood estimation with robust standard errors within the Mplus statistical software package [40]. The criterion variable--number of EBPs used in the past year--is treated as a count variable in the analyses by use of Poisson regression [41]. Count data are common in health services and implementation research, and statistical models to account for distributional characteristics of such data were addressed in our regression analyses that used the Poisson distribution [42-44]. Standard errors were adjusted to account for the clustering of respondents within communities (*k *= 17). Missing data were low (item covariance coverage >94%) and missing values for dependent variables were estimated using full information maximum likelihood (FIML) estimation [45,46]. Missing data in predictor variables excluded four participants, resulting in a final sample of 170 respondents used in the analyses.

**Figure 1:**
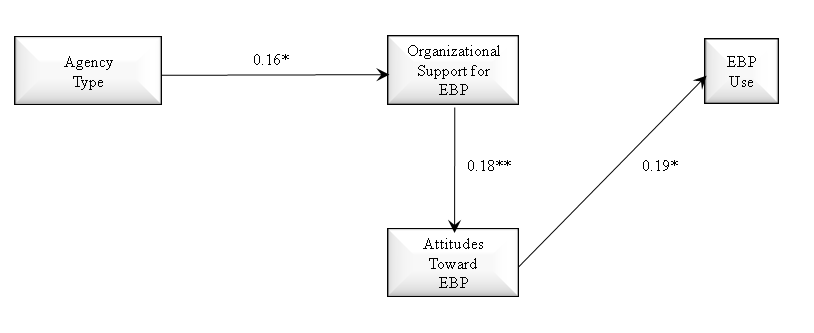
**Path model with full mediation effects of agency type on organizational support for evidence-based practice, provider attitudes toward evidence-based practice, and provider use of evidence-based practice**. N = 170; AIC = 2514.106, SBIC = 2513.678; *p < 0.05, **p < 0.01 (one-tailed).

**Figure 2:**
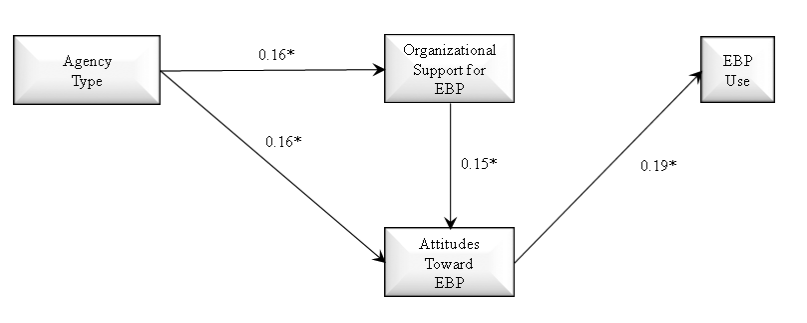
**Path model with partial mediation effects of agency type on organizational support for evidence-based practice and attitudes toward evidence-based practice, and effect of provider attitudes toward evidence-based practice on provider use of evidence-based practice**. N = 170; AIC = 2512.035, SBIC = 2511.577; *p < 0.05 (one-tailed).

**Figure 3:**
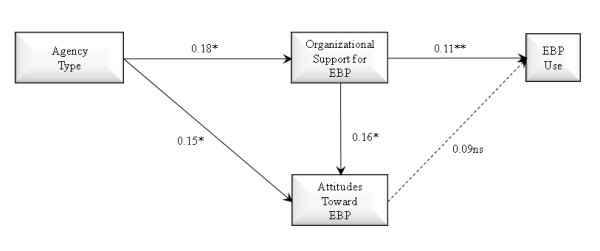
**Path model of partial mediation effects of agency type on organizational support for evidence-based practice and attitudes toward evidence-based practice, effect of organizational support for evidence-based practice on provider attitudes toward evidence-based practice, and effect of organizational support for evidence-based practice on provider use of evidence-based practice**. N = 170; AIC = 2437.127, SBIC = 2436.638; *p < 0.05, **p < 0.01 (one-tailed).

Three theoretically derived path models were tested and compared. Two mediational relationships were assessed: Whether organizational support for EBP mediates the association of agency type with attitudes toward EBP, and whether attitudes toward EBP mediate the association of organizational support for EBP and EBP use. Figure 1 shows a full mediation model in which the effect of agency type on provider attitudes toward adopting EBPs is fully mediated through organizational support for EBP (*i.e.*, no direct effect of organization type on attitudes toward EBP), with a final path from attitudes toward EBP to EBP use in practice. As shown in Figure 2, we estimated a partial mediation model with direct effects of agency type on both organizational support for EBP and provider attitudes toward adopting EBP, indirect effects of organization type on attitudes toward EBP mediated through organizational support, and subsequent effects of provider attitudes toward EBP on EBP use in practice. Figure 3 shows the same model but adds a path representing the direct effect of organizational support for EBP on EBP use.

Model fit was assessed using multiple indicators, including Akaike's Information Criterion (AIC) and sample size adjusted Bayesian Information Criterion (SBIC). In both cases, smaller values indicate better model fit [47,48]. The assessment of model fit was used to evaluate the fit of the overall hypothesized model to the data. We assessed Hypotheses five and six regarding partial mediation of organizational type by the level of organizational support for EBP, and mediation of organizational support on EBP use by comparing fit of the full mediation versus partial mediation models. The other study hypotheses were evaluated through an examination of the effect size and statistical significance of path coefficients. All path coefficients are standardized regression coefficients except for the paths linking antecedent variables with EBP use. These final path coefficients represent Poisson regression coefficients for which standardization is not appropriate. We utilized one-tailed significance tests for path coefficients in keeping with our directional hypotheses [49]. Employee characteristics including age, race, gender, education, job tenure, and years working in youth mental health services were entered as covariates to control for the potential influence of provider characteristics on provider attitudes.

### Common source bias

Consistent with recommendations by Podsakoff *et al. *[50], items that may potentially exhibit common source bias have proximal and methodological separation in that they are measured in different ways and in different substantive sections of the survey. One set of questions relates to respondent attitudes as measured on a Likert-like type scale, another set of questions assesses respondent use (or not) of individual EBPs, and the third set of questions assesses the presence or absence of nine different organizational behaviors in the past year. Each set of questions are embedded in a series of questions with a different substantive focus (*i.e.*, attitudes toward EBP, actual use of EBPs, and organizational characteristics related to EBPs). Additionally, several of the study's primary hypotheses involve objectively measured criterion such as type of agency (*i.e.*, public or private agency) which should exhibit minimal potential for systematic bias. We explored the use of analytical models to assess common source bias (*i.e.*, latent variables constructed of all potentially biased items), but the approach proved untenable given the small sample size and differing variable types (*i.e.*, categorical, count, continuous). Finally, to promote accurate and unbiased responses and minimize any social pressures or expectations, the survey was conducted voluntarily, confidentially, and online [50].

## Results

Table 1 provides descriptive statistics for the total sample and public and private sector participants. Consistent with expectations, the mean score for organizational support of EBPs and attitudes toward EBPs was lower in public sector organizations than in private sector organizations. Public and private sector organizations also differed by gender composition (public sector agencies had a lower percentage of women) and age (public sector agencies had a higher average age).

**Table 1:** Sample Characteristics

	Total (N = 170)			Public (n = 41)			Private (n = 129)			p
	Nominal Variables	Continuous Variables		Nominal Variables	Continuous Variables		Nominal Variables	Continuous Variables		
		
VARIABLE	%	Mean	SD	%	Mean	SD	%	Mean	SD	
Organization type										
Private	75.9									
Government	24.1									
Gender										
Female	72.9			61.0			76.7			
Male	27.1			39.0			23.3			< 0.05
Race										
Non-White	11.2			17.1			9.3			
White	88.8			82.9			90.7			
Education										
Some college	1.2			2.4			0.8			
Bachelor's degree	16.5			7.3			19.4			
Master's degree	69.4			75.6			67.4			
Doctoral degree	12.9			14.6			12.4			
Age (years)		40.8	11.2		43.9	11.4		39.8	11.0	< 0.05
Job tenure (years)		5.8	6.6		7.8	8.2		5.2	5.9	
Years in child mental health		9.6	7.9		11.2	8.4		9.1	7.7	
Organizational EBP support		3.9	2.6		3.2	2.4		4.1	2.6	< 0.05
EBPAS total score		2.8	0.5		2.6	0.6		2.8	0.5	< 0.05
EBP use		6.3	5.7		6.3	5.3		6.2	5.9	

Note: Sample size varied slightly within each group; Significant differences between Public and Private are noted in column *p*

Table 2 provides the correlation matrix for the study variables in which several bivariate correlations of interest are evident, including a positive association between level of organizational support and whether the provider was working in a private agency (r = 0.157, p < 0.05). Working in a private agency was also positively associated with more favorable attitudes toward adopting EBP (r = 0.190, p < 0.05). Some of the zero-order correlations were non-significant here, however, more complex relationships were next examined in the context of all study variables in the path analyses. As noted above, although some assumptions regarding preconditions for assessing effects of mediation of organizational support on EBP by provider attitudes were not evident in the correlation matrix, this mediation hypothesis was more directly explored in the path models below.

**Table 2:** Correlation matrix of demographic characteristic covariates, agency type, organizational support for EBPs, and Attitudes toward Evidence-Based Practice

	Male	White	Education	Age	Job tenure	Child MH	Private agency	Org. EBP support
Sex (male)								
Race (White)	0.006							
Education	0.047	0.043						
Age	0.059	0.129	0.289 ***					
Job tenure	0.165 *	0.050	0.094	0.546 ***				
Child MH	0.138	0.150	0.236 **	0.669 ***	0.727 ***			
Private agency	-0.152 *	0.106	-0.096	-0.158 *	-0.122	-0.118		
Org. EBP support	0.104	-0.012	-0.085	-0.005	0.064	0.004	0.157 *	
EBPAS total	-0.104	0.065	0.004	-0.074	-0.145	-0.069	0.190 *	0.149

Note: N = 170; Child MH = years providing children's mental health services; Org. EBP support = organizational support for EBP; EBPAS total = evidence-based practice attitude scale total score; *p < 0.05; **p < 0.01; ***p < 0.001

The three models to be tested are illustrated in Figures 1 through 3. Figure 1 illustrates a full mediation model of the effect of agency type on attitudes toward EBP mediated by organizational support for EBP, and full mediation of the effect of organizational support for EBP on EBP use mediated by attitudes toward EBP. Figure 2 illustrates a partial mediation model of the association of agency type on attitudes toward EBP with partial mediation through organizational support for EBP. Figure 2 also tests the direct association of attitudes toward EBP and EBP use. Figure 3 adds a test of partial mediation for the association of organizational support for EBP partially mediated by attitudes toward EBP. These are the three competing models of organization type and organizational support for EBP use as predictors of attitudes toward adopting EBP, and EBP use.

The model shown in Figure 2 demonstrates lower AIC and SBIC values relative tothe Figure 1 model, which indicates better fit for the partial mediation (Figure 2) model. This partial mediation model demonstrates significant direct effects of organization type on provider attitudes as well as indirect effects of organization type on provider attitudes toward EBP being mediated by level of organizational support for EBP. This model also shows a significant effect of attitudes toward EBP on EBP use. The final model in Figure 3 demonstrates lower AIC and SBIC values relative to the models in Figures 1 and 2 indicating the model in Figure 3 is the best fitting model. This model demonstrates the relationships consistent with those found in Figure 2 and shows an additional significant association between organizational support for EBP and EBP use. However, in this model the significant association between attitudes toward EBP and EBP use found in the model two, while in the expected direction, was no longer statistically significant. After assessing model fit we examined our primary hypotheses based on model three. Consistent with hypothesis one, we found that organization type had a significant direct effect on provider attitudes toward adopting EBP, with private agency providers endorsing more positive attitudes toward adopting EBP relative to those from governmental organizations (p < 0.05). As anticipated in hypothesis two, private organizations exhibited higher levels of support for EBP relative to governmental agencies (p < 0.05). Hypothesis three was also supported as indicated by the significant positive association between organizational support for EBP and provider attitudes toward adopting EBP (p < 0.05). Hypotheses four was not supported in the final model as the positive association between provider attitudes toward adopting EBPs and EBP use, while having a small effect size in the expected direction, was no longer statistically significant (p > 0.05). Hypothesis five was partially supported in that attitudes toward EBP acted as a mediator of the effect of agency type and organizational support in model two, while in the final model was in the expected direction, was no longer statistically significant. Hypothesis six was supported in that a higher level of organizational support for EBP was associated with greater EBP. Finally, hypothesis seven was not supported as the model did not support the mediational paths linking indirect effects of organizational support for EBP on EBP use through attitudes. As shown in Table 3, of the demographic variables, only job tenure was significantly associated with less positive provider attitudes toward adopting EBPs (p < 0.05). Finally, as shown in Figure 3, the effect sizes of the significant path coefficients indicate small but significant effects [51] in the hypothesized directions.

**Table 3:** Regression analysis of provider demographic characteristics on provider attitudes toward evidence-based practice (EBPAS Total Score).

	Full Mediation Model			Partial Mediation Model		
		
Characteristic	*B*	SE	*β*	B	SE	*β*
Male	-0.131	0.079	-0.11 *	-0.101	0.077	-0.09
White	0.080	0.140	0.05	0.047	0.142	0.03
Education	0.017	0.061	0.02	0.021	0.055	0.03
Age	-0.002	0.004	-0.05	-0.001	0.004	-0.02
Job tenure	-0.016	0.007	-0.20 *	-0.014	0.008	-0.18 *
Years in Child MH	0.008	0.009	0.12	0.006	0.009	0.10

Note: *B *= unstandardized regression coefficient; SE = standard error; *β *= standardized regression coefficient; *p < 0.05 (one-tailed)

## Discussion

This study demonstrates that organizational characteristics are related to EBP use in complex ways. First, organization type matters in regard to both organizational supports for EBP and provider attitudes toward adopting EBP. Providers working in private organizations endorsed more positive attitudes toward adopting EBP. Private organizations also provided more organizational support for EBP, leading to more favorable provider attitudes toward adopting EBP. Consistent with previous research, organizational support was also associated with uptake of a new technology [29], in this instance EBP. In addition, the findings are consistent with prior studies suggesting that public and private organizations will reflect their institutional environments in a predictable manner.

We anticipated a significant association between provider attitudes toward adopting EBP and EBP use. In model two, this association was significant, however in the final model, while the effect was in the hypothesized direction, the path was not statistically significant. While the larger study was not specifically designed to test this association, this is an area ripe for future study in that theory predicts that such relationships should occur. More targeted studies should be designed to more explicitly test these issues. This is important because gaining staff buy-in and having palpable organization support are believed to be important factors in effective implementation of innovation in organizations [29]. Our results suggest that in the absence of strong organizational support for EBP, attitudes are likely to play a greater role in the adoption and use of EBP in practice. This study provides support for the importance of organizational context in the uptake and use of EBP in mental health provider organizations [52], and provides initial empirical evidence validating proposed links between organizational support for innovation and attitudes towards innovation, and also between organizational support and use of innovation [6].

The results regarding organization type suggest that institutional differences persist despite the recent emphasis on making government organizations more competitive and responsive to changes in their environments. The 'new public management' movement has developed over the past several decades with a primary goal of 'reinventing government' to function similarly to private corporations [53]. The language of competition and adaptation may be becoming less alien to public sector organizations; however, this study shows that certain gaps still remain between public and private sector organizations regarding innovation and EBP implementation. Over time, these differences may diminish as changes--such as the shift towards treating public sector clients as 'customers'--increase pressure to adopt and adapt innovation and improvements in service delivery [54]. For the present, though, implementation planners should remain cognizant of the potential need for additional resources and attention to support successful adoption and usage of EBPs being sensitive to the nature of both public and private sector organizations.

The findings presented here beg the question: What can be done to facilitate organizational support for EBP? Recent literature suggests some promising directions. First, the literature from business and management suggests that organizational processes and communications can emphasize the importance of innovation implementation [55] (in this case, EBP) and create a more positive organizational climate for implementation. In mental health services, this could include marketing EBP not only to organizations, but to consumers of services. Indeed, emphasizing the efficacy and effectiveness of EBP in improving the lives of mental health service consumers gets at the core purpose of mental health services. Because many providers enter their chosen field in order to help others, this appeal may be particularly consistent with their sense of what is important for them and for consumers [56].

Second, leadership at multiple levels in organizations can affect staff perceptions. In a recent implementation study, leadership and organizational support were cited by agency managers and providers as critically important in acceptance and use of the EBP [56,57]. Of particular importance is 'first-level' leadership of direct organizational and clinical supervisors for line staff. First-level leaders are those in most contact, and likely to have the greatest influence on direct service providers [58]. However, clear and consistent messages of support for EBP from top management (*e.g.*, agency executive directors), middle management, program managers, and clinical supervisors are important in creating a positive implementation climate [29]. Consistent and positive messages supporting learning and use of EBP can help to create a culture conducive to excellence in service provision.

## Strengths and limitations

An important strength of this study is that it replicates a previously found relationship between organizational type and provider attitudes toward EBP using a more geographically diverse sample of organizations in 17 community service settings across the United States. The measure of organizational support for EBP, developed in previous evaluation work and utilized in the present study, appears to have good utility for the study of organizational supports for EBP, attitudes towards EBP, and use of EBP. Further developmental and psychometric work should be undertaken to better elucidate the reliability and validity of the measure. However, as noted above, we found good unidimensional scale structure and good internal consistency, and there are appropriate analytic methods for handling count data. Some limitations of the present study should also be noted. First, the study was cross-sectional and causal inferences cannot be drawn based on the data and analyses presented here. However, the directions of effects are consistent with theory, somewhat mitigating this concern. Second, additional dimensions that may vary across organizational such as size, client case-mix, measures of bureaucracy, and staff self-selection processes could not be accounted for in our analyses, so the specific mechanisms creating the public-private EBP differences remains unclear. Future research should incorporate these factors into the analyses of organizational EBP adoption and implementation dynamics within public and private organizations. Third, our measure of organizational support assessed the number of different types of EBP support provided, whereas it might prove useful to assess additional dimensions of support, such as their frequency and intensity. Thus, future work should examine how different measures of organizational support for EBP may relate to provider attitudes towards EBP and EBP use. Fourth, mean EBP use scores were similar in public and private sector organizations. This could be because of larger contextual influences on providers or because of differences in directives (rather than support). Fifth, all variables were based on respondent self-reports. While organization type is likely to be objective, common method variance might have influenced the results presented here, even though the scales and measures were structured differently from each other and most attempted to assess specific observable behaviors. Finally, EBP use was determined by provider self-report; however, we took a conservative approach by only counting EBP use for those practices for which training had been received by each respondent.

## Summary

The organizational supports for EBP identified in the present study may provide some guidance for agency directors and administrators, but by no means provides a complete compendium of strategies for improving the climate for EBP implementation. For example, providing local trainings or in-services on-site may facilitate attendance by clinical staff. Behavioral health organizations should also attend to the literature on transfer of training that informs methods of training most likely to result in actual use of new knowledge and skills in practice [59]. In-vivo practice and ongoing coaching and/or consultation hold promise for effective implementation. In addition to merely the presence of support for EBP, the intensity and ways in which support is communicated are likely to be important in affecting attitudes towards and use of EBP. Rather than hopeful or even ardent messages about the value and importance of EBP, intentional and targeted marketing of EBP to providers and consumers may help to drive the uptake of EBP in both public and private agencies [60].

Finally, enhancing system and organizational context in general [61], and for implementation climate [55] in particular, can encourage providers to consider EBP as a viable way to improve services for consumers with mental health needs. This does not imply that we should encourage providers to tacitly accept EBP. Rather, a practical-scientist approach [62] that promotes critical appraisal of client mental health need and the best methods to address such need should be taken. Such an approach should be consistent with definitions of EBP that consider research evidence, clinical expertise and judgment, and consumer choice, preference, and culture [63,64]. By proceeding in a way that takes a balanced consideration of values and needs of systems, organizations, providers, and consumers, we can endeavor to implement and provide high quality care for those with mental health needs.

## Competing interests

The authors declare that they have no competing interests.

## Authors' contributions

GA contributed to the theoretical background and conceptualization of the study, was the developer of the EBPAS, and contributed to the survey design, writing, data analysis, and editing. DS contributed to the theoretical background and conceptualization of the study, and contributed to the writing, data analysis, and editing. CW contributed to the theoretical background and conceptualization of the study, was responsible for survey design and data collection, and contributed to the writing and editing.

All authors have read and approved this manuscript.

## Appendix 1: Evidence-Based Practices Survey

Instructions: We are now going to ask you a few questions about specific evidence-based practices that you may or may not be familiar with. Please answer each question for each EBP included below. Have you used the EBP in the past year with children and families participating in the system of care program in your community?

Response options included: Yes, No, Don't Know

1. Anger Coping/Anger Management

2. Antidepressants for Mood Disorders

3. Assertiveness Training

4. Behavior Modeling

5. Behavior Therapy

6. Behavioral Parent Training

7. Behavioral Teacher Training

8. Brief Strategic Family Therapy

9. Case Management

10. Cognitive Behavioral Therapy

11. Common Sense Parenting

12. Coping Cat Program for Anxious Youth

13. Emotive Imagery Therapy

14. Family Education and Support

15. Functional Family Therapy

16. Incredible Years Program - Webster-Stratton

17. Interpersonal Therapy for Adolescents

18. Mentoring

19. Multisystemic Therapy

20. Parent-Child Interaction Therapy

21. Positive Behavioral Supports

22. Problem Solving Skills Training

23. Rational Emotive Therapy

24. Relaxation Training

25. Respite

26. Self-control Instruction Training

27. Social Skills Training

28. Stimulant Medication for ADHD

29. Systematic Desensitization

30. Therapeutic Foster Care

31. Wraparound
